# Bullet impacts in building stone excavate approximately conical craters, with dimensions that are controlled by target material

**DOI:** 10.1038/s41598-022-22624-z

**Published:** 2022-10-21

**Authors:** Oliver Campbell, Tom Blenkinsop, Oscar Gilbert, Lisa Mol

**Affiliations:** 1 School of Earth and Environmental Sciences, Cardiff University, Cardiff, CF10 3AT UK; 2 Department of Geography and Environmental Management, University of the West of England, Bristol, BS16 1QY UK

**Keywords:** Geology, Planetary science, Mechanical properties

## Abstract

Bullet impacts are a ubiquitous form of damage to the built environment resulting from armed conflicts. Bullet impacts into stone buildings result in surficial cratering, fracturing, and changes to material properties, such as permeability and surface hardness. Controlled experiments into two different sedimentary stones were conducted to characterise surface damage and to investigate the relationship between the impact energy (a function of engagement distance) and crater volumes. Simplified geometries of crater volume using only depth and diameter measurements showed that the volume of a simple cone provides the best approximation (within 5%) to crater volume measured from photogrammetry models. This result suggests a quick and efficient method of estimating crater volumes during field assessments of damage. Impact energy has little consistent effect on crater volume over the engagement distances studied (100–400 m), but different target materials result in an order of magnitude variation in measured crater volumes. Bullet impacts in the experiments are similar in appearance to damage caused by hypervelocity experiments, but crater excavation is driven by momentum transfer to the target rather than a hemispherical shock wave. Therefore in contrast to predictions of impact scaling relationships for hypervelocity experiments, target material plays the dominant role in controlling damage, not projectile energy.

## Introduction

Contemporary conflicts cause devastating damage to the built environment through the use of aerial bombings, artillery strikes, and ground based weapons. In addition to the large scale destruction imposed by explosives and artillery, smaller scale damage results from bullet and shrapnel impacts. This scale of damage is often overlooked during initial post-conflict surveys of damaged heritage, despite being common to nearly all current and historical conflicts since the use of early firearms. Many buildings damaged this way are considered to be culturally significant heritage sites, such as religious buildings across Ukraine damaged by artillery and shrapnel during the current conflict^1^, or the targeted demolition and looting of Palmyra in Syria^2^.

There is an emerging understanding that for stone buildings, these regularly overlooked forms of damage are associated with more than just surficial cratering^3–8^. Fracture networks can extend deep within the stone, creating 4-7 times more new surface area than the impact crater alone^8^. Grain fracturing and pore space collapse directly below the impact lead to compaction, locally reducing permeability and surface hardness. This volume is surrounded by a region of greater surface hardness reduction and increased permeability^7^. Internal fracture intensity decreases with distance away from the crater floor, which, together with the surface hardness and permeability changes, affects the stone’s resistance to further deterioration from weathering processes^8,9^.

A higher effective porosity, i.e. the combination of inherent porosity and impact induced fractures, facilitates greater ingress of moisture via capillary flow^10^. This moisture can dissolve matrix and constituent minerals, reducing overall stone strength and further increasing its effective porosity. Moisture transports dissolved salts into the stonework, which create outward pressures upon crystallisation, widening pore spaces and fractures. This results in the loss of material from the surface of the stone, reduced stone strength, and an exacerbated negative feedback loop of stone deterioration^10–13^. It is thus vital for effective conservation efforts that the surface and subsurface expressions of impact damage are comprehensively understood. This study characterises impact damage under controlled conditions for different target materials and projectiles in order to investigate potential relationships with resultant damage.

Digital imaging can be used to observe and document damage, and to generate 3D models for digital preservation^14^. For heritage affected by armed conflict, the capture of adequate digital imagery for representative 3D models may not be possible in all situations, so alternative methods must be used. Campbell et al.^15^ compared crater profiles measured manually using a Barton comb with profiles extracted from a 3D model. This study investigates a simpler approach: can crater volumes be estimated using just depth and diameter measurements and simplified volume geometries? A simple approach for estimating crater volumes is invaluable for surveys of heritage damage in conflict zones, where factors such as safety or accessibility can limit effective time on site.

Comparing crater volumes to the kinetic energy of the impactor allows important deductions to be made about the physics of the cratering mechanism. In the latter part of the paper, accurate crater volume estimates from photogrammetry are used to compare the damage and scaling relationships of bullet impacts with those of hypervelocity experiments. The comparison yields insights into cratering mechanics.

## Methods and materials

### Target materials and projectile impacts

**Figure 1:**
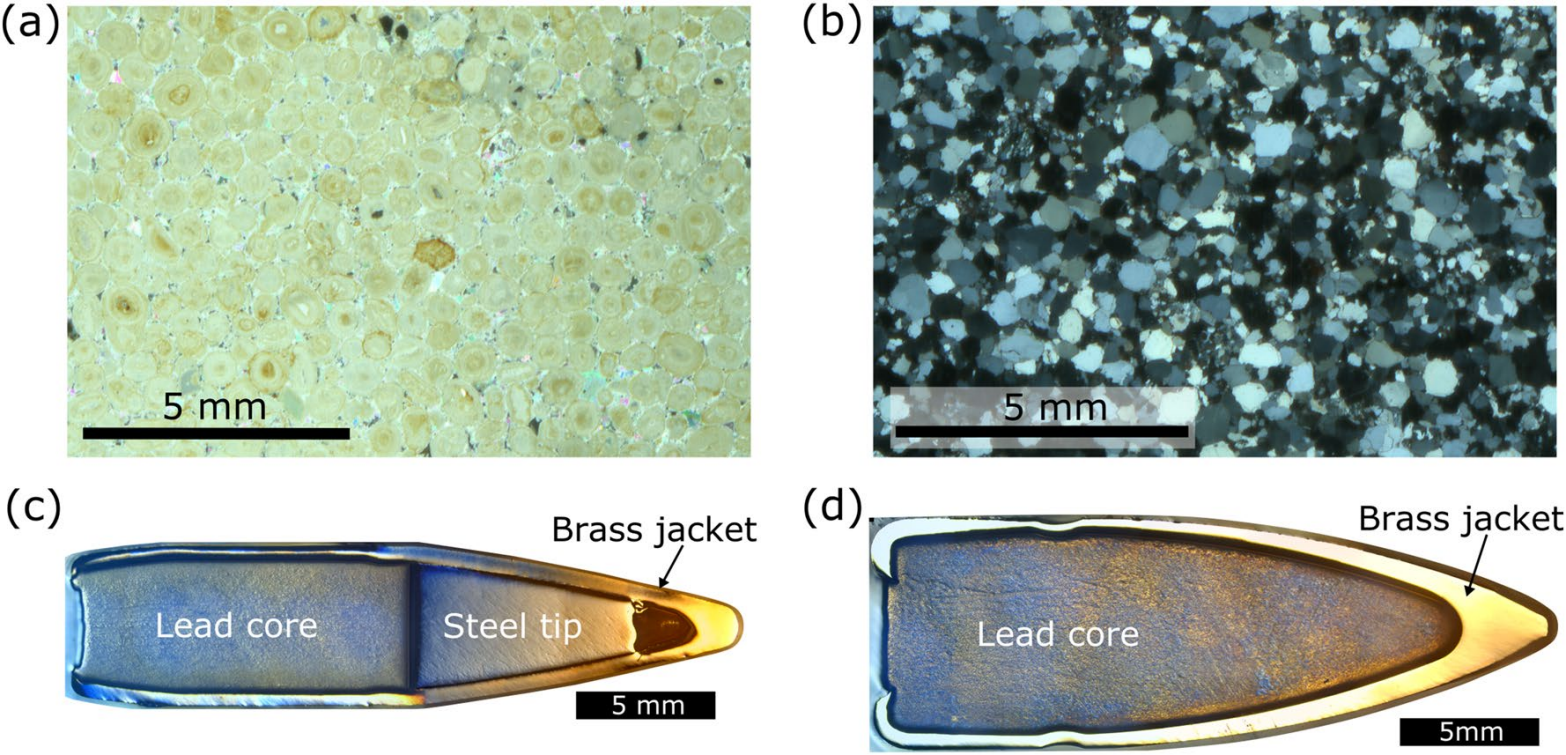
(**a**) Cross polarised photomicrograph of Cotswold Hill Cream Limestone. (**b**) Cross polarised photomicrograph of Stoneraise Red Sandstone. (**c**) Reflected light image of a cross section through a $5.56\,\hbox {x}\,45\,\hbox {mm}$ NATO projectile. (**d**) Reflected light image of a cross section through a $7.62\,\hbox {x}\,39\,\hbox {mm}$ projectile. (From^15^).

Freshly quarried cubes ($15\,\hbox {x}\,15 \hbox {x}\,15\,\hbox {cm}$) of Stoneraise Red Sandstone (SRS) and Cotswold Hill Cream Limestone (CHCL) were selected as target stones because of their analogous properties to heritage stones in the Middle East, such as the Mokattam Limestone of Egypt, and the Umm Ishrin sandstones of Petra, Jordan^16–18^. The Cotswold Hill Cream Limestone is an oolitic grainstone from the Middle Jurassic Inferior Oolite (quarried near Ford, UK). The average grain size is $0.5\,\hbox {mm}$ and it has a porosity of $\sim$20% (see Fig. 1a). The Stoneraise Red Sandstone has a fine-medium ($0.125-0.5\,\hbox {mm}$) grain size, and comes from a quartz rich bed from the Permian New Red Sandstones (quarried near Penrith, U.K.) (see Fig. 1b). It has a porosity of $\sim$11% and generally no internal layering, though some blocks exhibit visible beds of coarser grains ($\sim 1\,\hbox {mm}$). The density of each sample was determined by measuring the dry mass of the block and dividing by the volume ($3375\,\hbox {cm}^3$ for all samples).

Controlled firearm experiments were carried out at Cranfield Ordnance Test and Evaluation Centre (Gore Cross, UK) to simulate conflict damage to stone. Two different types of ammunition used in contemporary and past conflicts were fired at $90^{\circ }$ to the target face. Firstly, $5.56\,\hbox {x}\,45\,\hbox {mm}$ NATO (abbreviated as NATO) is a standardised cartridge used in the British SA80 assault rifle, the American M16 family of assault rifles, and many other military issue firearms around the world. The second ammunition type is a $7.62\,\hbox {x}\,39\,\hbox {mm}$ cartridge (abbreviated as AK-47), commonly fired from AK-variant rifles, such as the widely known AK-47. Both ammunition types are a spitzer ogive nosed projectile with a brass jacket and lead core (see Fig. 1c,d), but the NATO projectile also has a steel tip within the brass jacket. The AK-47 projectile has a mass of 7.95 grams (123 grains) and a bulk density of $13.25\,\hbox {g}\hbox {cm}^{-3}$. The NATO projectile has a mass of 4.04 grams (63 grains) and a bulk density of $8.08\,\hbox {g}\hbox {cm}^{-3}$. The bulk density of each projectile was calculated by dividing the projectile mass by the volume of water displaced by the projectile in a graduated cylinder.

Both cartridges were remotely fired from mounted proof barrels 14 m from the target. Projectile velocity was measured using a Weibel SL-525P Doppler radar system ($400\,\hbox {mW}$, $10.525\,\hbox {GHz}$). The kinetic energy ($E_k$) of the projectile at the point of impact was calculated using:

1 $$\begin{aligned} E_k=\frac{1}{2}mv_i^2 \end{aligned}$$

where *m* is the projectile mass and $v_i$ is projectile velocity at the point of impact. Test shots were conducted on an open range at standard propellant load to measure the velocity decay of each projectile, providing desired velocities for simulated engagement distances. Propellant loads for each cartridge were adjusted to reduce velocities to simulate impacts at distances of $200\,\hbox {m}$ in limestone and sandstone targets. Further experiments at a simulated distance of $400\,\hbox {m}$ were conducted in limestone targets to acquire a set of damaged blocks for a different study, but whose crater geometry is beneficial to include here. One further shot was conducted at full propellant load (muzzle velocity) into a sandstone target. Average engagement distances (i.e. the distance between combatants) of urban firefights during the Iraq War ranged from $26\,\hbox {m}$ to over $126\,\hbox {m}$, and most soldiers are trained for engagement distances of 0–$600\,\hbox {m}$, so $200\,\hbox {m}$ represents a reasonable distance for simulating impacts in both urban and open scenarios^19,20^. Concrete blocks were placed on all faces, except the target face, for confinement. Target blocks with bedding were oriented so that foliations were parallel to the target face (i.e. perpendicular to trajectory). Natural stone is typically strongest when loaded perpendicular to bedding, so target blocks were oriented with a consistent bedding orientation relative to the target face.

### Target properties

To investigate the influence of target strength on impact damage, compression tests were conducted on undamaged blocks of each stone type to measure the uniaxial compressive strength (UCS)^21^ and the indirect tensile strength^22^. Cylindrical cores ($20\,\hbox {mm}$ diameter x $40\,\hbox {mm}$ length) were drilled perpendicular and parallel to bedding. Cores were loaded at a constant rate of $0.005\,\hbox {mm}\hbox {s}^{-1}$ using a Zwick/Roell Z050 static testing machine. The standard force, deformation, and time step were recorded using the TestXpert III software (version 1.5). Linear regression was carried out on straight sections of the stress-strain curves to find the axial Young’s modulus parallel and perpendicular to bedding for each stone type. The UCS ($\sigma _u$) was calculated using the equation:

2 $$\begin{aligned} \sigma _u=\frac{P}{A_c} \end{aligned}$$

where *P* is the failure load and $A_c$ is the cross sectional area of the core.

Further cylindrical cores ($30\,\hbox {mm}$ diameter) for measuring the indirect tensile strength were cut parallel to bedding, and then into $15\,\hbox {mm}$ thick disks for Brazilian tests^22^. The prepared disks were mounted on their thin edge between flat plates and loaded perpendicular to bedding at a constant rate of $0.005\,\hbox {mm}\hbox {s}^{-1}$. The indirect tensile strength ($\sigma _t$) was then calculated by:

3 $$\begin{aligned} \sigma _t=2\frac{P}{\pi tD_d} \end{aligned}$$

where *P* is the failure load, *t* is the thickness of the disk and $D_d$ is the disk diameter.

The ultrapulse velocity (UPV) was measured in twelve undamaged blocks of each stone type using a Proceq Pundit 200 with $54\,\hbox {kHz}$ exponential transducers (pulse voltage = $200\,\hbox {V}$, receiver gain = x1, frequency = $20\,\hbox {Hz}$). UPV was measured in each of the three orthogonal directions by placing the transducers on opposite faces. A bulk UPV value was calculated by averaging the three orthogonal directions.

### Characterising damage morphology

Damaged samples were photographed through a $360^\circ$ rotation at three overlapping camera positions using a 14-megapixel Fujifilm FinePix S3400 digital camera. Samples were overturned and the process was repeated, resulting in a total of 6 overlapping camera orientations. Additional images were taken across the impact crater to ensure adequate capture of morphology. Meshroom (v2020.1.1), a free and open-source structure from motion (SfM) pipeline developed by AliceVision®, was used to process the $\sim$300-400 images into a 3D mesh^23,24^. In CloudCompare (version (2.11.3)^25^), the impact crater was isolated from the full block mesh, scaled, and oriented with the target surface horizontal and an azimuth direction of $000^\circ$ directed towards the top edge of the block in its firing position.

Crater volumes were measured in CloudCompare and morphology profiles extracted using a Python code (version 3.8.11) from 3D point clouds (code available in^15^). Impact craters were outlined in QGIS (version 3.16.15) from plan view photographs. The edge of the crater was defined visually as the transition point from a depression, not including radial fractures, to undamaged target face. These outlines were analysed in ImageJ (version 1.53) to measure the crater area (*A*), which was used to calculate an area equivalent diameter ($D_eq$) using:

4 $$\begin{aligned} D_{eq}=2\sqrt{\frac{A}{\pi }} \end{aligned}$$

Crater volumes measured from the digital models were compared to the volumes of three simplified geometries (*V*) derived from just crater depth (*d*) and radius ($r = 0.5D_{eq}$) measurements. The simplified geometries selected have previously been used to describe crater geometries in hypervelocity experiments: a simple cone^26–28^ where $V=\frac{1}{3}\pi r^2d$ , a spherical cap^29^ where $V=\frac{1}{6}\pi d(3r^2+d)$ , and a parabaloid, typically representing the transient crater^27^ where $V=\frac{1}{2}\pi r^2d$ .

## Results

### Target properties

Compression tests show that the sandstone targets have higher compressive and tensile strengths than the limestone targets. Reported strengths are the average value of *n* number of cores measured ± one standard deviation (also available in Supplementary Table S1). The uniaxial compressive strength perpendicular and parallel to bedding for the Stoneraise Red Sandstone (SRS) (*n*=9) is 40.0 ± $5.9\,\hbox {MPa}$ and 44.0 $\pm \,13.1\,\hbox {MPa}$ respectively , while the Cotswold Hill Cream Limestone (CHCL)(*n*=9) values are 10.6 $\pm \,1.5\,\hbox {MPa}$ and 8.8 $\pm \,2.1\,\hbox {MPa}$ respectively. The indirect tensile strength parallel to bedding (i.e. loading direction perpendicular to bedding) for the SRS (*n*=10) is 5.0 $\pm \,0.3\,\hbox {MPa}$ and 2.2 $\pm \,0.2\,\hbox {MPa}$ for the CHCL (*n*=12). SRS samples have a higher axial Young’s Modulus with 2.6 $\pm \,0.4\,\hbox {GPa}$ and 3.0 $\pm \,0.6\,\hbox {GPa}$ parallel and perpendicular to bedding respectively. CHCL (*n*=9) has values of 1.5 $\pm \,0.3\,\hbox {GPa}$ and 1.1 ± $0.5\,\hbox {GPa}$ for the same orientations. SRS (*n*=12) has an average UPV of $833\,\hbox {ms}^{-1}$, faster than the average of $569\,\hbox {ms}^{-1}$ in CHCL (*n*=12) targets.

## Surface damage

**Figure 2:**
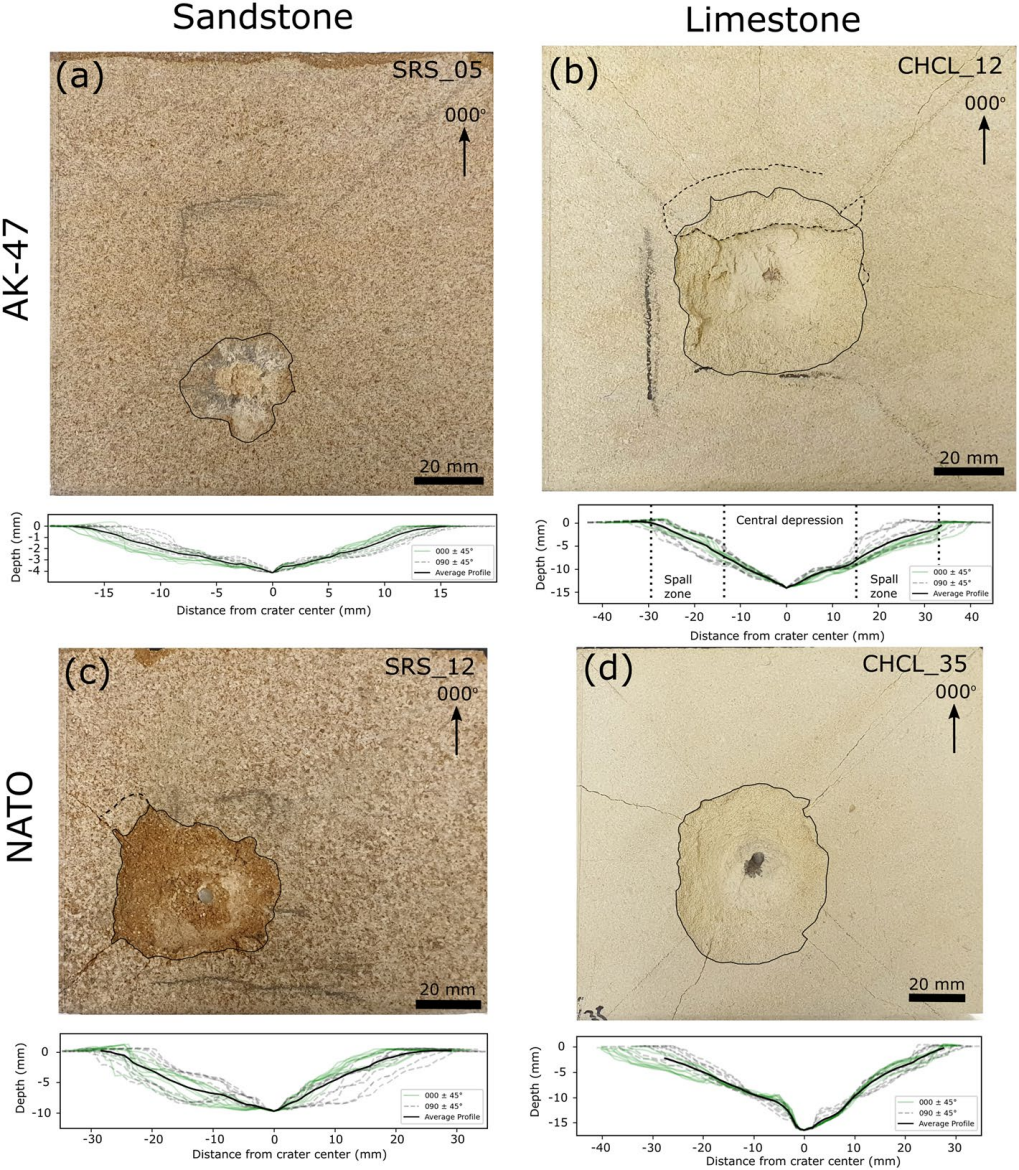
Photographs of impact craters and summary of 18 cross section profiles caused by $7.62\,\hbox {x}\,39\,\hbox {mm}$ (AK-47) (**a**,**b**) and $5.56\,\hbox {x}\,45\,\hbox {mm}$ NATO (NATO) (**c**,**d**) projectiles. An azimuth direction of $000^\circ$ points towards the top edge of the target block in its firing position. Profiles oriented between $000^\circ \,\pm \,045^\circ$ are coloured green, while profiles oriented $45^\circ$ either side of $090^\circ$ are dashed grey. The crater outline is marked with a solid black line and incipient spall fragments by a dashed black line. Fractures can be seen radiating from the impact crater to the edge of the target block. The steel tip of the NATO projectile can be seen embedded in the target block (**c**,**d**).

All experiments resulted in the formation of an impact crater and material loss. The floor of the impact craters have a fine grained, powdery appearance with a pale discolouration. Damage varies with lithology and projectile type. Sandstone targets impacted with AK-47 projectiles exhibit shallow, cone-shaped craters with average depths of 4.6 mm, diameters of $33.8\,\hbox {mm}$, and volumes of $1.9\,\hbox {cm}^3$ (see Table 1). There are few visible surface fractures surrounding the impact crater and where present they are short and have closed apertures. Some samples have a dark grey discolouration in and around the impact crater from lead within the projectile (see Fig. 2a). Limestone targets have a more complex, two-part structure of a deep central depression surrounded by a shallow dipping spall region (see Fig. 2b). Of the four samples, three had impact velocities of 429 $\pm \,5\,\hbox {ms}^{-1}$ and one of $532\,\hbox {ms}^{-1}$. The slower impacts had average depth and diameter measurements of $14.0\,\hbox {mm}$ and $59.8\,\hbox {mm}$ respectively. The faster impact had had larger values of $42.5\,\hbox {mm}$ and $101.9\,\hbox {mm}$ respectively. The crater volumes, measured from photogrammetry models, show the difference in dimensions between the slower and faster impacts, with the three slow impacts having an average volume of $14.1\,\hbox {cm}^3$ and the fast impact $107.6\,\hbox {cm}^3$.

**Table 1:** Summary of average depth (*d*), diameter ($D_{eq}$), measured volume (*V*), and depth/diameter ratio for targets of Stoneraise Red Sandstone and Cotswold Hill Cream Limestone shot with $7.62\,\hbox {x}\,39\,\hbox {mm}$ (AK-47) and $5.56\,\hbox {x}\,45\,\hbox {mm}$ NATO projectiles.

	Sandstone		Limestone	
	AK-47	$\hbox {NATO}^\mathrm{a}$	$\hbox {AK}-47^\mathrm{b}$	NATO
d ($\hbox {mm}$)	4.6	12.5	14.0	23.3
$D_{eq}$ ($\hbox {mm}$)	33.8	47.3	59.8	65.1
*V* ($\hbox {cm}^3$)	1.9	8.3	14.1	24.7
$d/D_{eq}$	0.13	0.23	0.23	0.35
Number of samples	4	5	3	4

$^\mathrm{a}$Averages do not include sample SRS_23 which had an impact velocity considerably faster than other samples. For SRS_23: $d\,=\,17.5\,\hbox {mm}$, $D_{eq}$ = $68.5\,\hbox {mm}$, $V\,=\,24.6\,\hbox {cm}^3$, $d/D_{eq}$ = 0.25.

$^\mathrm{b}$Averages do not include sample CHCL_09 which had an impact velocity considerably faster than other samples. For CHCL_09: $d\,=\,42.5\,\hbox {mm}$, $D_{eq}$ = $102.2\,\hbox {mm}$, *V* $=\,107.6\,\hbox {cm}^3$, $d/D_{eq}$ = 0.42.

**Figure 3:**
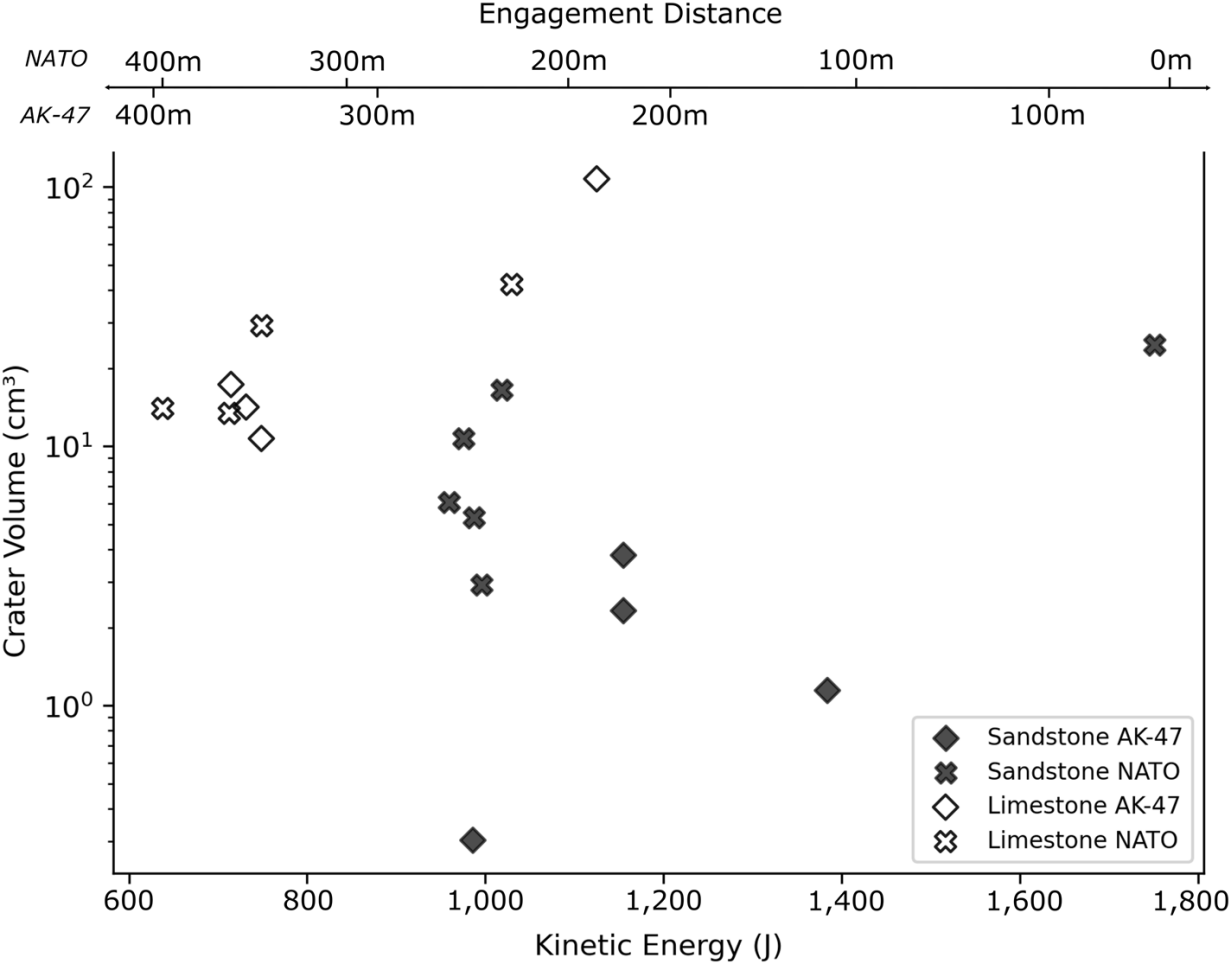
Photogrammetric crater volume against the kinetic energy of projectiles, $7.62\,\hbox {x}\,39\,\hbox {mm}$ (AK-47) and $5.56\,\hbox {x}\,45\,\hbox {mm}$ NATO, at impact. Engagement distance is derived from the projectile velocity for a given kinetic energy.

The NATO projectile, excluding the test conducted at full propellant load, produced deeper ($12.5\,\hbox {mm}$), wider ($47.3\,\hbox {mm}$), and larger volume ($8.3\,\hbox {cm}^3$) craters in the sandstone targets than the AK-47 projectiles. The test conducted at full propellant load had the largest diameter ($68.5\,\hbox {mm}$) and volume ($24.6\,\hbox {cm}^3$) of the 6 samples, but it was not the deepest crater. The steel tip of the NATO projectile remained embedded in the floor of the impact crater in 5 out of 6 experiments (see Fig. 2c,d). Crater profiles are more complex than the simple cone-shaped craters created by AK-47 projectiles, with a shallow spall zone surrounding a steep sided central excavation. Fractures with open apertures radiate from the impact crater, and can reach the edge of the target face. Limestone targets have more radial fractures with wider apertures than impacts into sandstone targets. The craters have a two-part structure of steep sided central excavation and shallow dipping spall zone. NATO impacts into limestone targets caused craters with an average depth of $23.3\,\hbox {mm}$ and diameter of $65.1\,\hbox {mm}$. Crater volumes are over twice as large (24.7 vs. $11.0\,\hbox {cm}^3$) as comparable impacts into sandstone targets. For the studied engagement distances (i.e. simulated distance between firearm and target), the impact energy does not appear to have a strong influence on crater volume. For near identical impact energy, there can be up to an order of magnitude difference in crater volume (see Fig. 3).

**Figure 4:**
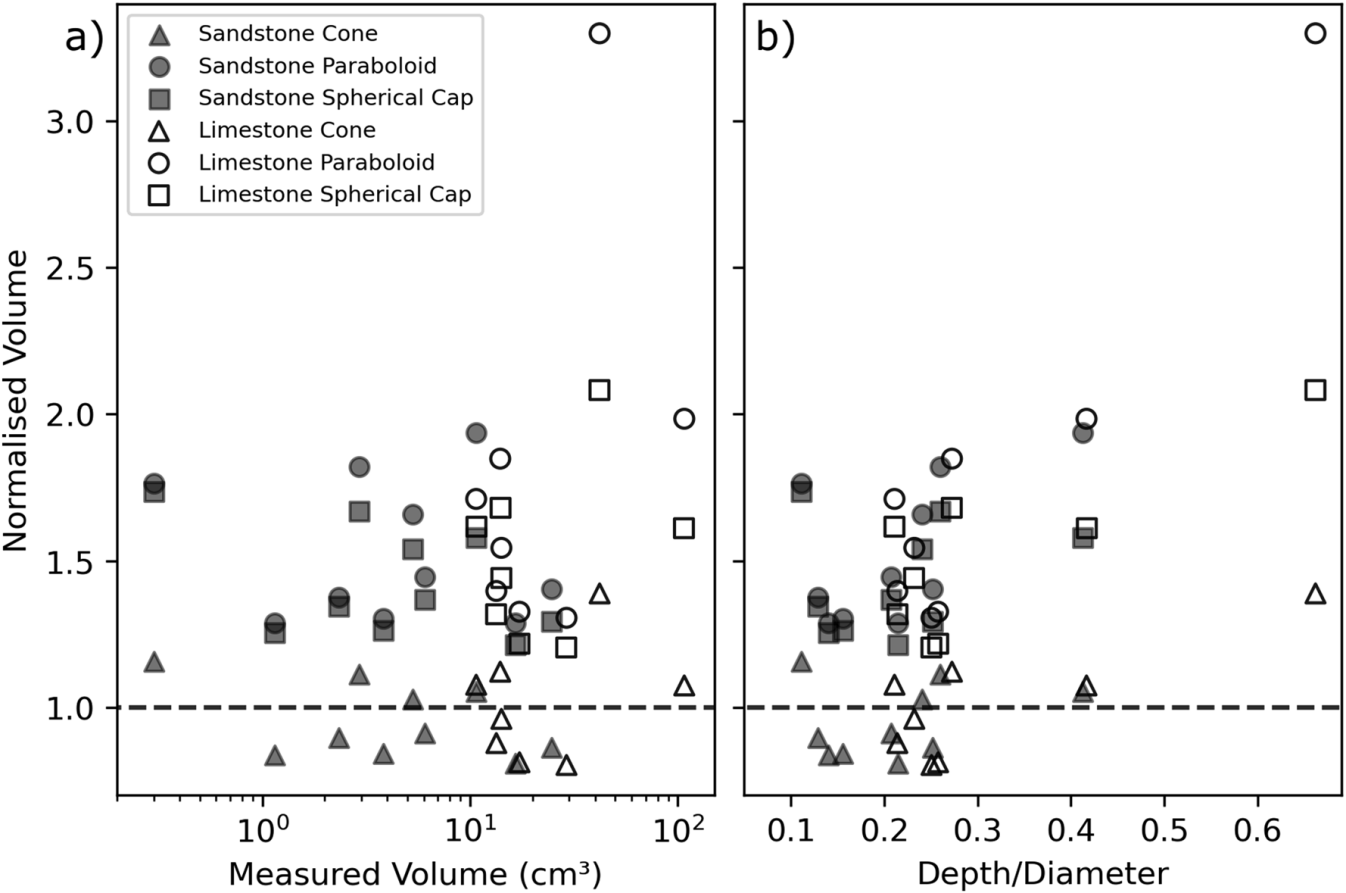
(**a**) Estimated crater volumes normalised to the crater volume measured from photogrammetry models plotted against photogrammetric volume. Sandstone targets (filled markers) have smaller crater volumes than limestone targets (hollow markers). The simple cone geometry (triangle marker) provides the closest estimate to the measured volume (dashed line). (**b**) Estimated crater volumes normalised to the crater volume measured from photogrammetry models plotted against depth/diameter ratio. There is a statistically significant, though weak, trend of increasing overestimation with increasing depth/diameter ratio (see Supplementary Table S2).

Of the studied simplified crater geometries, the simple cone provides the closest estimate to the volume of the crater measured by photogrammetry, with sandstone craters underestimated 4.9% ± 12.0 on average and limestone craters slightly overestimated by 1.4% ± 18.2. These values are substantially smaller than the overestimation for sandstone and limestone craters by the spherical cap (52.8% ± 23.2 and 80.2% ± 61.2 respectively) and paraboloid (42.6% ± 17.9 and 52.1% ± 27.4 respectively) geometries (see Fig. 4a). The simple cone geometry was also applied to asymmetric craters created by oblique impacts^15^. The geometry estimates crater volumes within 6.3% of the photogrammetry values, almost as accurate as for the perpendicular impacts (4.9%).

## Discussion

For both the simple cone-shaped crater and the more complex two-part structures, radial fractures centred on the impact crater, and crushed target material on the crater floor, resemble damage resulting from hypervelocity experiments^28,30,31^. In this study, relatively undeformed projectile material (steel tip of NATO projectile) is embedded in the floor of the crater, unlike most hypervelocity experiments in which the projectile is melted and/or ejected^32,33^. The embedded projectile material here lies at the base of short, cylindrical penetration channels, akin to observations made from experiments investigating the penetration of rigid steel rods into concrete^34^. Corrosion of the projectile’s steel tip when exposed to the elements over some time after impact may locally exacerbate fractures, similar to the deterioration seen in reinforced concrete due to corrosion of rebar, except on a much smaller scale^35^. There is no evidence of any AK-47 projectiles penetrating into targets, only smearing of lead material around or in the impact crater.

**Figure 5:**
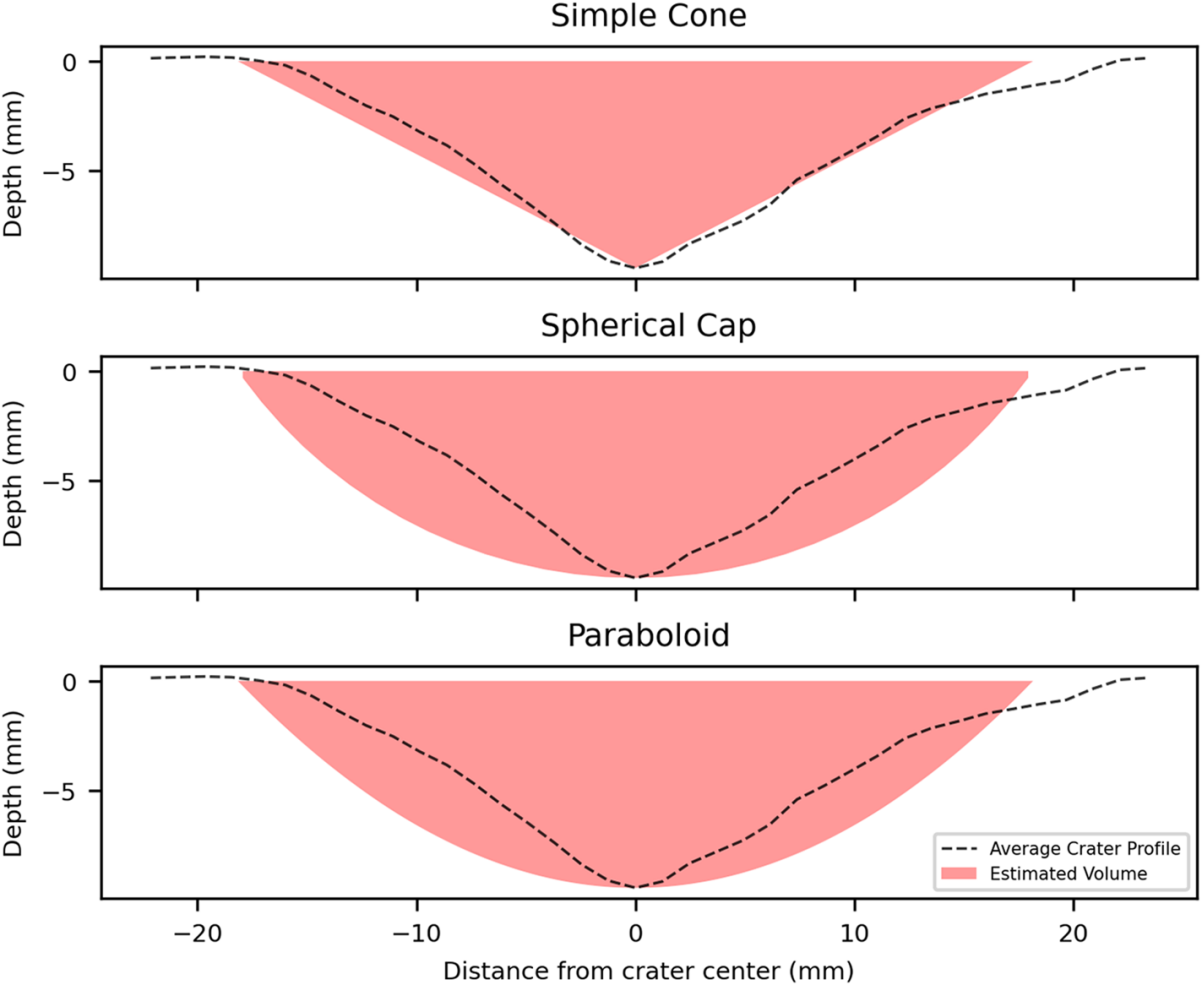
Average cross section profile of sample SRS_14 and cross section through the simplified crater geometries that use the max depth and $D_{eq}$.

The simple cone geometry provides the best estimation (within 5%) of the measured crater volume using depth and diameter measurements. The spherical cap and paraboloid geometries substantially overestimate the measured crater volume. This overestimation stems from the morphological differences of the geometries, visualised in Fig. 5. The concave down form of crater walls, created by the two part structure of a deep central pit and surrounding spall zone profiles, diverges from the simplified geometries (cone, spherical cap, paraboloid) which have a straight or concave up form to wall profiles. This effect is more prominent in the spherical cap and paraboloid geometries, which is reflected in overestimation of 50–80%. Additional geometry measurements, such as the width and depth of the central excavation or spall zone, may provide better estimates of crater volume, but the extra time and effort required in measuring these values would compromise the goal of a quick and efficient field method.

Simplifying crater geometries to estimate volume from two rapidly acquired measurements allows many impacts to be studied in a shorter time than photogrammetry. Measurements of depth and diameter are possible with simple analogue tools such as calipers and depth gauges. Although this study took a digital approach to these measurements, it is unlikely the substitution with analogue values will affect the overall conclusions, as Campbell et al.^15^ show reasonable agreement between analogue crater profiles obtained using a Barton comb and profiles measured from photogrammetry models. Volumes can be estimated in the field with the simplified geometry, providing an overview of crater volume distribution while investigators are on site, supporting first-response assessments of conflict damage to heritage. Imaging of a site for photogrammetry models can be done relatively quickly (minutes per impact), but the post-field production and analysis of models (hours to tens of hours) lengthens the overall method time. Smartphone cameras, and the Light Detection and Ranging (LiDAR) capability of new generation iPhones or hand-held scanners, are increasingly able to generate 3D SfM models approaching the precision of those using digital cameras and SfM software, or those derived from terrestrial laser scanning (TLS)^36,37^. The LiDAR sensors in iPhones were developed to enhance photographs, and not to produce surface coordinates like traditional TLS. However, downloadable applications have been developed to utilise the iPhone hardware to produce models that are of comparable precision to SfM and TLS methodologies^36^. At present, the measurement of crater volumes and fracture orientations from 3D models in the field is still limited by the need for computers with appropriate software. Analogue field measurements remain the simplest and most accessible means of initial damage assessment.

Photogrammetry and simplified volume estimations could be viewed as complimentary methods. Volume estimation from depth and diameter measurements provides a good first order method of quantifying impact damage and its distribution, enabling on site testing of hypotheses and targeted data collection towards areas at highest risk of future deterioration. If the situation permits, imaging of the site for SfM photogrammetry models provides a more accurate quantification of the damage, as well as digitally preserving heritage sites in a way that can be used as a baseline to track changes over time^38,39^.

The three simplified geometries presented here show an increasing overestimation of crater volume with increasing depth/diameter ratio (see Fig. 4b). This is likely the result of the deeper central pits, causing divergence of crater wall morphology from the straight or concave up profile of the simplified geometries. Therefore care should therefore be taken when estimating the volume of craters with higher depth/diameter ratios. This method has been developed for impact craters with good rotational symmetry (created by perpendicular impacts), however the simple cone geometry does suitably estimate the volume of craters created by oblique impacts (within 6.3%).

**Figure 6:**
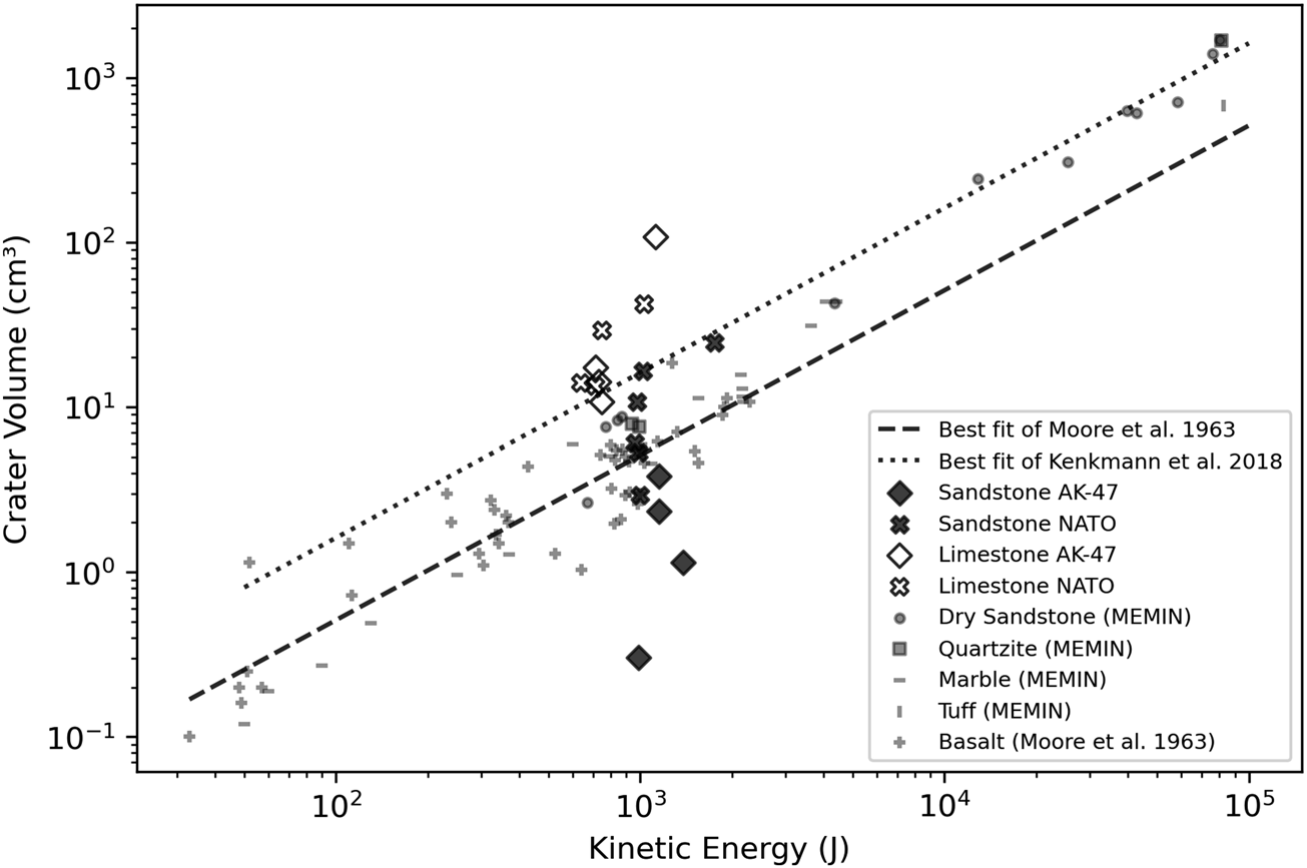
Plot showing the trend of increasing crater volume with increasing kinetic energy (at impact) displayed by hypervelocity experiments from Moore et al.^44^ and the Multidisciplinary Experimental and Modelling Impact Crater Research Network (MEMIN)^43^. The results of this study have a wider range of crater volumes for the narrow range of impact energies studied, particularly impacts into sandstone with $7.62\,\hbox {x}\,39\,\hbox {mm}$ (AK-47) projectiles. NATO = 5.56 x $45\,\hbox {mm}$ NATO projectile.

In hypervelocity experiments, crater volume is linked to the kinetic energy of the projectile (i.e. impact energy). The greater the amount of energy available, the larger the peak pressures experienced by the target, and the greater the material failure^40–42^. Hypervelocity experiments exhibit well established correlations between increasing impact energy and crater volume (Fig. 6). Impact energies and crater volumes presented here are of a similar magnitude to some hypervelocity experiments (Fig. 6). However, for the range of impact energies (approximating engagement distances of $100\,-\,400\,\hbox {m}$) of this study, the crater volumes do not follow the relationship with impact energies observed in the MEMIN (Multidisciplinary Experimental and Modelling Impact Research Network)^43^ or Moore et al.^44^ hypervelocity studies. For a given impact energy, limestone targets from this study have larger crater volumes than hypervelocity experiments, whereas sandstone targets impacted by AK-47 projectiles have smaller volumes. No systematic relationship between crater volume and impact energy is evident.

To compare impact experiments into targets with different properties, it is useful to use dimensionless parameters. Holsapple^41^ gave a generalised equation for crater volume in strength-dominated (i.e. the scale of impact means crater formation processes are governed by material strength), non-porous materials:

5 $$\begin{aligned} V \propto \frac{m}{\rho _t}*(\frac{\rho _t v_i^2}{Y})^\frac{3\mu }{2}*\frac{\rho _t}{\rho _p} ^{1-3v} \end{aligned}$$

where *V* is the crater volume, *m*, $\rho _p$ and $v_i$ are the projectile’s mass, density and velocity, $\rho _t$ is the target density, *Y* is the measure of target strength, and $\mu$ and *v* are scaling exponents^45^. For strength controlled craters, *V* increases at a rate somewhere between momentum scaling ($V\propto mv_i$) and energy scaling ($V\propto mv_i^2$), imposing limits for $\mu$ of: 1/3 $<\,\mu \,< 2/3$^45,46^. Equation 8 can also be written using three scaling parameters (pi-scaling): cratering efficiency ($\pi _v$), a strength term ($\pi _3$), and a density term ($\pi _4$):

6 $$\begin{aligned} \pi _v \propto \pi _3^\frac{-3\mu }{2}*\pi _4^{1-3v} \end{aligned}$$

Multiple linear regression of the experiments conducted here failed to produce values for $\mu$ and *v* of any statistical significance and within the limits for $\mu$. The creation of the generalised equation for non-porous materials poses the question of its applicability to the porous targets of this study. However, hypervelocity impact experiments with a range of non-zero sample porosities could be used to calculate values of $\mu$ and *v*^43,44,47^, whilst numerical models found no change in $\mu$ for target porosities 0-35%^48^. This suggests that target porosity is not the sole reason for the failure to obtain values of $\mu$ and *v* in this study.

The use of pi-scaling assumes that the impact causes a shock wave that is equivalent to an explosion at depth, and assumes a point source^47^. The validity of this assumption may be why hypervelocity impact craters remain relatively circular except at very low impact angles^47^. A condition of the point source assumption is that impact velocity far exceeds the target sound speed^49^. The impact velocities of the experiments reported here are similar or below the UPV (i.e. sound speed) values of the target lithology, so these experiments may not produce a shock wave at impact. Without a shock wave, crater excavation is instead driven by momentum transfer from the projectile to the target, a process influenced by the strength of both the target and projectile materials. Limestone targets in this study had compressive and tensile strengths 75-80% and 50% weaker respectively than the sandstone targets, resulting in greater crater volumes than sandstone impacts, even at lower impact energies (see Fig. 3).

The strengths of each target lithology were measured under quasi-static strain rates ($<,10\,\hbox {s}^{-1}$), but rock strength is strain rate dependent, increasing rapidly after a threshold strain rate^50^. Rae et al.^51,52^ show that the dynamic compressive strength of rocks can be double the quasi-static strength at stain rates $>10^2\,\hbox {s}^{-1}$. Cho et al.^53^ show that tensile strength increases at strain rates $10^0$–$10^1\,\hbox {s}^{-1}$. Bullet impacts exhibit strain rates of $10^3$–$10^6\,\hbox {s}^{-1}$, varying due to quantities such as target and projectile material, impact energy, impact trajectory, and projectile shape^54–56^. The target strengths used here are therefore a minimum value. The clear correlation between target strength and crater volume indicates that any increase in strength due to strain rate may be comparable between the two lithologies.

The projectile strength in these experiments appears to have an influence on damage, with the harder steel tip of the NATO projectile resulting in larger impact craters than comparable impacts using the lead cored AK-47 projectiles. The steel tip of the NATO projectiles remains relatively undeformed and embedded in the crater floor, likely experiencing a greater interaction time with the target. Barnouin-Jha et al.’s^57^ low velocity (85–$250\,\hbox {ms}^{-1}$) experiments yielded results incompatible with proposed crater scaling relationships, which was suggested to have been due to increased interaction time between projectile and target. They propose that the penetration time is critical to the cratering process, and that depth/diameter ratios will be larger than expected for impacts at much higher velocities. Kenkmann et al.^43^ reported depth/diameter ratios ranging from 0.1 to 0.56 for impact velocities of 2500–$7850\,\hbox {ms}$. Average depth/diameter ratios of experiments here (0.13- 0.35) fall within this range of values for much lower impact velocities, so do not initially appear to support Barnouin-Jha et al.’s^57^ suggestion. The ogival shape of the projectiles in this study is different from the spherical projectiles used in both the hyper- and low velocity experiments discussed, possibly increasing penetration potential and reducing the direct comparability between the sets of experiments.

Target lithology is a bigger determining factor of final crater volume than impact energy, despite the scatter observed here (see Fig. 6)^15^. This could be used in conjunction with knowledge of heritage construction materials to prioritise post-conflict efforts on weaker materials. There is up to an order of magnitude variation amongst the crater volumes measured from photogrammetry models for the same impact energy (see NATO projectile into sandstone targets in Fig. 3). The cause of this variability in impact geometry under very similar impact conditions may be the result of internal variations within target lithologies. Despite target blocks being quarried from the same beds and oriented in the same way with respect to internal foliation, natural sedimentary stone has inherent variability that may result in variable crater volumes for the same conditions. There is similar inherent variability in hypervelocity experiments (e.g. MEMIN^43^ and Moore et al.’s^44^ data, see Fig. 6), for which scaling relationships could still be derived. Some different form of scaling relationships might exist for the ordnance velocity experiments presented here, which additional experiments at a greater range of impact energies could help to derive.

## Conclusions

Bullet impacts into limestone produce wider, deeper, and more voluminous impact craters than the same projectiles impacting sandstone targets. Limestone targets also have tensile strength 50% lower, and compressive strength values 75% lower than sandstone targets. Sandstone targets impacted with $7.62\,\hbox {x}\,39\,\hbox {mm}$ (AK-47) projectiles have shallow, cone-shaped craters. Targets impacted with $5.56\,\hbox {x}\,45\,\hbox {mm}$ NATO projectiles, and impacts of both projectiles into limestone targets, have a two part-structure consisting of steep sided central excavation pit surrounded by a shallow dipping spall zone. Radial fractures are centred around the impact and reach the edge of the target block, providing conduits and entry points for weathering agents such as salt and moisture.

The volume of a simple cone, calculated from two simple measurements of crater depth and diameter, estimates crater volume within 5% of the accurate value determined from photogrammetry models. This result allows for a quick and efficient method for initial assessment of heritage sites damaged in armed conflict.

Impact craters generated here are similar in size and morphology to craters generated by hypervelocity experiments. However, projectile velocities below the sound speed of the target, penetration of the projectile, and the lack of scaling between crater size and impact energy, imply that damage is not governed by a shock wave. Crater excavation is instead controlled by momentum transfer, strongly influenced by target and projectile properties. Thus over the range of impact energies studied, engagement distance has little consistent effect, but target material typically creates an order of magnitude variation in crater volume. This suggests that heritage sites built of stone with lower strength values are at risk of greater damage from conflict.

## Supplementary Information

Supplementary Information 1.

Supplementary Information 2.

## Data Availability

All the data used in this study is provided in the supplementary information.
