# Supplementary material for: Bullet impacts in building stone excavate approximately conical craters, with dimensions that are controlled by target material
Source: Sci Rep. 2022 Oct 21;12:17634. doi: 10.1038/s41598-022-22624-z (PMC9587296; doi:10.1038/s41598-022-22624-z)
Supplement: Supplementary file 2 — Supplementary Information 2. [file 41598_2022_22624_MOESM2_ESM.docx]

|  | | **Sandstone** | | ***n*** | **Limestone** | | ***n*** |
| --- | --- | --- | --- | --- | --- | --- | --- |
|  | **Bulk UPV (ms^-1^)** | 833 | | 12 | 569 | | 12 |
| **Perpendicular to bedding** | **UCS (MPa)** | 40.0 | ± 5.9 | 9 | 10.6 | ± 1.5 | 9 |
|  | **Axial Young’s Modulus (GPa)** | 2.6 | ± 0.4 | 9 | 1.5 | ± 0.3 | 9 |
| **Parallel to bedding** | **UCS (MPa)** | 44.0 | ± 13.1 | 9 | 8.8 | ± 2.1 | 9 |
|  | **Tensile (MPa)** | 5.0 | ± 0.3 | 10 | 2.2 | ± 0.2 | 12 |
|  | **Axial Young’s Modulus (GPa)** | 3.0 | ± 0.6 | 9 | 1.1 | ± 0.5 | 9 |

Supplementary Table S1: Summary of the mechanical properties of Stoneraise Red Sandstone (SRS) and Cotswold Hill Cream Limestone (CHCL). Bulk UPV is the average value of ultrapulse velocities (UPV) measured in each orthogonal direction. *n* = number of samples tested to give average strength value.

| **Lithology** | **Geometry** | **R^2** | **Coefficient** | **std error** | **P>\|t\|** |
| --- | --- | --- | --- | --- | --- |
| Sandstone | Simple Cone | 0.873 | 3.9223 | 0.5 | 0 |
|  | Spherical Cap | 0.906 | 6.4462 | 0.693 | 0 |
|  | Paraboloid | 0.873 | 5.8834 | 0.749 | 0 |
| Limestone | Simple Cone | 0.908 | 2.8341 | 0.341 | 0 |
|  | Spherical Cap | 0.974 | 5.4195 | 0.337 | 0 |
|  | Paraboloid | 0.908 | 4.2512 | 0.511 | 0 |

Supplementary Table S2: Summary of linear regression statistics between the depth/diameter ratio and the normalised crater volumes from simplified geometries.
